# Geographic disparities in COVID-19 testing and outcomes in Florida

**DOI:** 10.1186/s12889-022-14450-9

**Published:** 2023-01-11

**Authors:** Md Marufuzzaman Khan, Agricola Odoi, Evah W. Odoi

**Affiliations:** 1 Department of Public Health, College of Education, Health, and Human Sciences, University of Tennessee, Knoxville, TN USA; 2 Department of Biomedical and Diagnostic Sciences, College of Veterinary Medicine, University of Tennessee, Knoxville, TN USA

**Keywords:** COVID-19, Disparities, Spatial Clusters, Florida, USA

## Abstract

**Background:**

Understanding geographic disparities in Coronavirus Disease 2019 (COVID-19) testing and outcomes at the local level during the early stages of the pandemic can guide policies, inform allocation of control and prevention resources, and provide valuable baseline data to evaluate the effectiveness of interventions for mitigating health, economic and social impacts. Therefore, the objective of this study was to identify geographic disparities in COVID-19 testing, incidence, hospitalizations, and deaths during the first five months of the pandemic in Florida.

**Methods:**

Florida county-level COVID-19 data for the time period March-July 2020 were used to compute various COVID-19 metrics including testing rates, positivity rates, incidence risks, percent of hospitalized cases, hospitalization risks, case-fatality rates, and mortality risks. High or low risk clusters were identified using either Kulldorff’s circular spatial scan statistics or Tango’s flexible spatial scan statistics and their locations were visually displayed using QGIS.

**Results:**

Visual examination of spatial patterns showed high estimates of all COVID-19 metrics for Southern Florida. Similar to the spatial patterns, high-risk clusters for testing and positivity rates and all COVID-19 outcomes (i.e. hospitalizations and deaths) were concentrated in Southern Florida. The distributions of these metrics in the other parts of Florida were more heterogeneous. For instance, testing rates for parts of Northwest Florida were well below the state median (11,697 tests/100,000 persons) but they were above the state median for North Central Florida. The incidence risks for Northwest Florida were equal to or above the state median incidence risk (878 cases/100,000 persons), but the converse was true for parts of North Central Florida. Consequently, a cluster of high testing rates was identified in North Central Florida, while a cluster of low testing rate and 1–3 clusters of high incidence risks, percent of hospitalized cases, hospitalization risks, and case fatality rates were identified in Northwest Florida. Central Florida had low-rate clusters of testing and positivity rates but it had a high-risk cluster of percent of hospitalized cases.

**Conclusions:**

Substantial disparities in the spatial distribution of COVID-19 outcomes and testing and positivity rates exist in Florida, with Southern Florida counties generally having higher testing and positivity rates and more severe outcomes (i.e. hospitalizations and deaths) compared to Northern Florida. These findings provide valuable baseline data that is useful for assessing the effectiveness of preventive interventions, such as vaccinations, in various geographic locations in the state. Future studies will need to assess changes in spatial patterns over time at lower geographical scales and determinants of any identified patterns.

**Supplementary Information:**

The online version contains supplementary material available at 10.1186/s12889-022-14450-9.

## Background

The coronavirus disease 2019 (COVID-19) pandemic, caused by the novel severe acute respiratory syndrome Coronavirus 2 (SARS-CoV-2), is currently the most serious challenge to global health. In the U.S, the first COVID-19 infections were reported in the West Coast [1], after which the disease rapidly spread to the Northeastern followed by the Southeastern parts of the country [2–4]. The U.S. has recorded more cases and deaths than any other country, with 3,472,234 confirmed cases, 175,924 hospitalizations, and 129,584 deaths as of July 15, 2020 [5].

The spread of the disease to Southeastern U.S. is particularly concerning due to the higher prevalence of several pre-existing health conditions that places many individuals in this region at higher risk of both COVID-19 infection and more severe outcomes compared to those in the rest of the country [6, 7]. The higher prevalence of comorbidities in Southeastern U.S. may be attributable to large proportions of older (> 65 years old) residents as well as large rural and minority populations with limited access to preventive healthcare [8–10]. Moreover, there is evidence that the risk factors for COVID-19 are disproportionately distributed geographically, with a tendency to cluster in certain areas defined by racial/ethnic, rural and socioeconomic characteristics [11]. This may result in disparities in COVID testing, higher incidence of disease and more adverse health outcomes in some locations compared to others [12–16]. Identifying communities that are at significantly higher risk of the disease and severe outcomes can help guide policies around testing and COVID-19 prevention and control. It can also provide baseline data to better understand the spatial dynamics of the pandemic and help public health officials gauge how mitigation efforts have impacted the disease burden in different populations across the U.S. Therefore, the objective of this study was to identify disparities in COVID-19 testing, incidence/cases, hospitalizations, deaths, and case fatality rates in Florida using the data of the first five months of the pandemic that were available at the time of analysis.

## Materials and methods

### Ethics approval

This study was reviewed and approved by the University of Tennessee Institutional Review Board (IRB). The IRB number is UTK IRB-20–06,105.

### Study design, area, and population

**Fig. 1:**
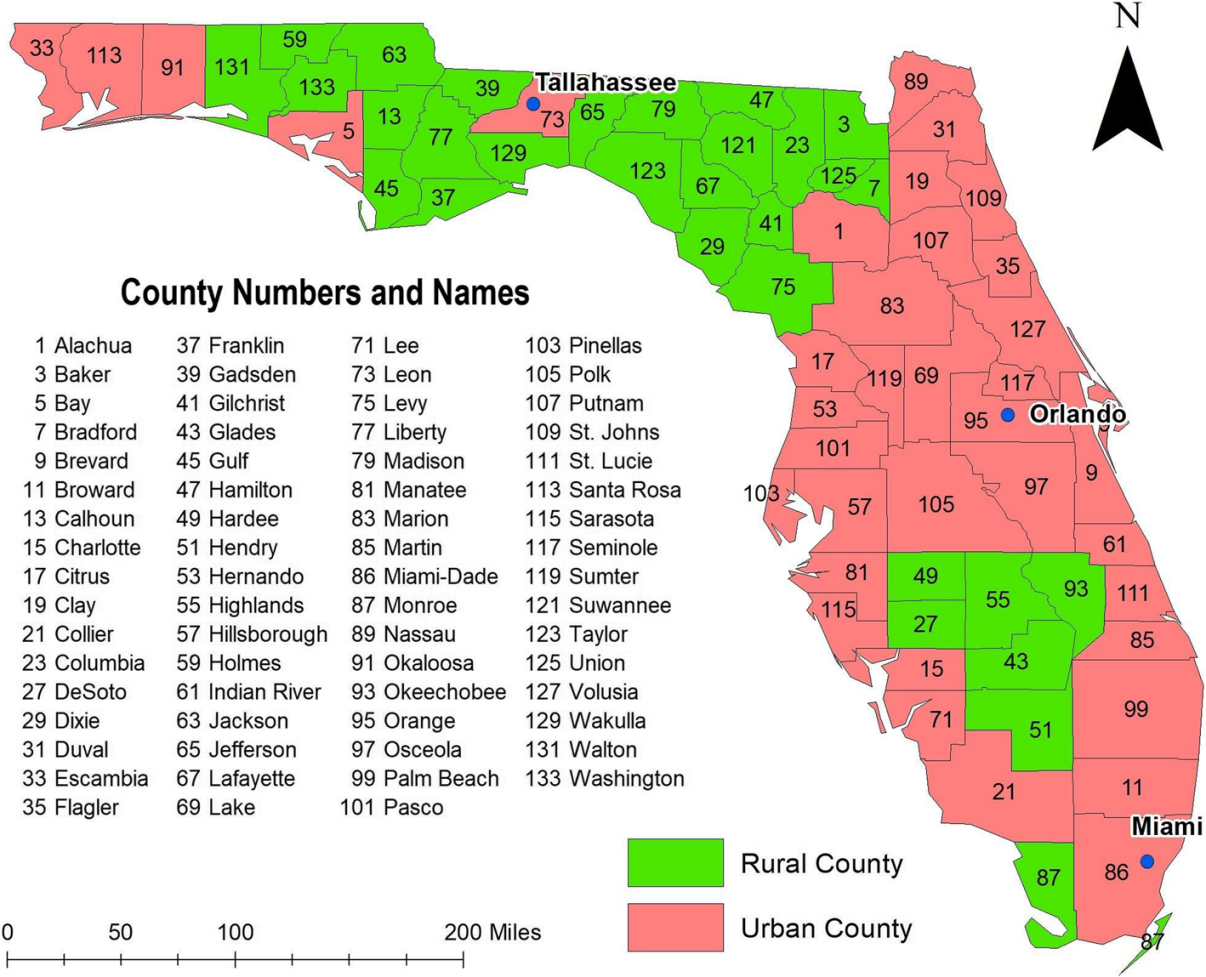
Rural/urban classification of Florida counties

This is a retrospective ecological study conducted at the county level in the state of Florida using publicly available COVID-19 data collected from March 1 to July 15, 2020. Florida is the 3^rd^ most populous state in the U.S., with approximately 20.9 million people distributed as follows; 49% male, 22.3% (0–19 years old), 57.7% (20–64 years old), and 20% (≥ 65 years old) (Florida Department of Health, 2019). The majority (77.4%) of the population are White, 16.9% are Black, and all other races comprise 5.7% of the population. By ethnicity, 25.7% of the population is Hispanic-Latino and the rest is non-Hispanic [17]. These demographic characteristics foreshadow the demographic changes predicted for the U.S. population by 2050 [18]. Approximately 44% of the counties in Florida are classified as rural (Fig. 1), with Miami-Dade being the most urban (0.4% rural population) and most densely populated county (1430/square mile), and Lafayette in North Central Florida being the most rural (100% rural population) and the most sparsely populated (16/square mile) county [19, 20].

### Data sources

County-level data for total number of: persons who tested positive for COVID-19, persons who tested negative for COVID-19, hospitalized cases, and deaths among Florida residents were extracted from Florida Department of Health (DOH) COVID-19 dashboard [21]. Total number of tests was defined as the sum of number of persons with positive and negative COVID-19 tests. Population estimates for 2018 for each of the 67 counties were obtained from Florida Population Atlas [22]. County-level cartographic boundary shape files were downloaded from the United States Census Bureau TIGER Geodatabase [23].

### Data preparation and descriptive analysis

The following COVID-19 metrics were computed cumulatively for the study period for each county:

- (a) Testing rate = no. COVID-19 tests/2018 county population*100,000;
- (b) Positivity rate = no. positive COVID-19 tests/all people tested in the county*100;
- (c) Incidence risk = no. COVID-19 cases/all people at risk in the county (i.e. 2018 county population) *100,000;
- (d) Percent of hospitalized COVID-19 cases = no. COVID-19 cases hospitalized/no. COVID-19 cases in the county*100;
- (e) Hospitalization risks = no. COVID-19 cases hospitalized/all people at risk in the county (i.e. 2018 county population) *100,000;
- (f) Case-fatality rates = no. COVID-19 deaths/no. COVID-19 cases in the county*100;
- (g) COVID-19 mortality risks = no. COVID-19 deaths/all people at risk in the county (i.e. 2018 county population) *100,000.

All descriptive analyses-including mean, standard deviation, median, lower quartile, upper quartile, and interquartile range were performed in SAS 9.4 [24]. All variables were assessed for normality using the Shapiro–Wilk test. Means and standard deviations were used to summarize normally distributed variables, while medians and lower and upper quartiles were used to summarize non-normally distributed variables.

### Cluster analyses

Clusters of testing rates and positivity rates were investigated using Kulldorff’s circular spatial scan statistics (CSSS), a spatial epidemiological tool for detecting and identifying circular clusters. This was implemented in SaTScan version 9.6 [25]. A discrete Poisson probability model specifying circular non-overlapping high or low risk purely spatial clusters was used. The circular window size was set at 13.5% of the population at risk. This window size was selected based on the population of Miami-Dade County, which has the largest population in Florida. This window size was selected to ensure that all spatial units, including the largest unit (i.e. Miami-Dade County), had a chance to be identified as a cluster. The likelihood ratio test and 999 Monte Carlo replications were used for statistical inference. Clusters were considered significant if the p-value for the relative risk was less than or equal to 0.05. Only low-risk clusters with relative risk ≤ 0.8 and high-risk clusters with relative risk ≥ 1.2 were considered meaningful [26].

Clusters for COVID-19 outcomes were investigated using Tango’s flexible spatial scan statistics (FSSS), a spatial epidemiological tool for detecting and identifying circular and irregularly shaped high-risk clusters. This was implemented in FleXScan v 3.1.2 [27, 28]. Poisson probability models with a restricted log likelihood (LLR) ratio (specifying an alpha of 0.2) and a maximum cluster size of 15 counties were specified to preclude potential inclusion of counties with non-elevated estimates of outcome variables. For statistical inference, 999 Monte Carlo replications were used and statistical significance was assessed using a critical *p*-value of 0.05.

### Cartographic displays

QGIS version 3.22.0 was used to display the geographic distribution of all COVID-19 metrics and the location of spatial clusters [29]. Jenk’s optimization classification scheme was used to determine critical intervals for displaying the geographic distribution of COVID-19 metrics as choropleth maps.

## Results

### Testing rates

**Fig. 2:**
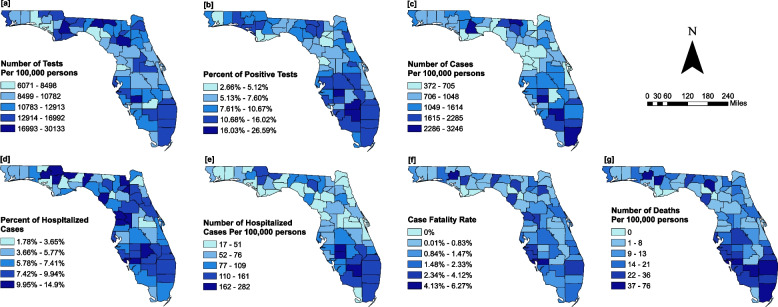
Geographic distribution of COVID-19 (**a**) testing rates, (**b**) positivity rates (**c**) incidence risks (**d**) percent of hospitalized cases (**e**) hospitalization risks (**f**) case fatality rates and, (**g**) mortality risks in Florida

The median testing rate for the state of Florida was 11,697 tests per 100,000 persons, and testing rates varied from 6,071 to 30,133 per 100,000 persons across the state (Fig. 2a, Table 1). Counties with testing rates above the state median were concentrated in North Central, Central, and Southeastern Florida while those with testing rates below the state median were concentrated in Northwest and Central Florida (Fig. 2a). The lightest colors represent low testing rates while dark blue colors represent high testing rates.

**Table 1:** Summary statistics for various COVID-19 outcome variables

**Outcome variable**	**Mean**	**SD**^a^	**Median**	**Lower quartile**	**Upper quartile**	**IQR**^b^
Number of tests/100,000 persons	12,473.25	3831.50	11,697.47	10,091.51	14,059.53	3968.00
Percent of positive tests^c^	9	4	9	6	12	6
Number of cases/100,000 persons	1178.87	659.71	877.95	726.86	1446.45	719.59
Percent of hospitalized cases^d^	7	3	7	5	9	4
Number hospitalized cases/100,000 persons	82.82	50.84	66.02	45.05	104.33	59.27
Case fatality rate (%)	1	1	1	1	2	1
Number of deaths/100,000 persons	16.34	15.06	11.71	5.39	23.86	18.46

^a^Standard deviation

^b^Interquartile range

^c^Computed as: (number of persons with positive tests)/(number of persons with positive tests + number of persons with negative test)*100

^d^Normal variable

**Fig. 3:**
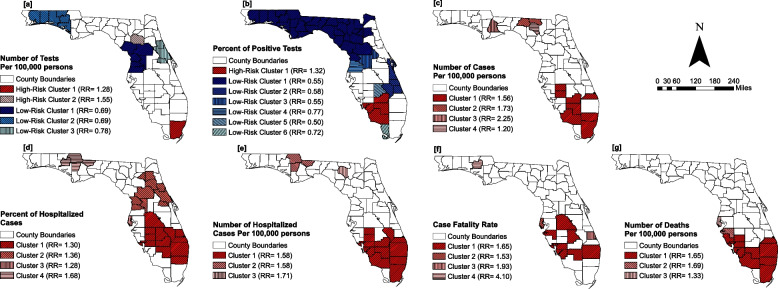
Geographic distribution of high or low risk clusters of COVID-19 (**a**) testing rates, (**b**) positivity rates (**c**) incidence risks (**d**) percent of hospitalized cases (**e**) hospitalization risks (**f**) case fatality rates and, (**g**) mortality risks in Florida

Two clusters of high testing rates and three clusters of low testing rates were identified (Fig. 3a). High testing rate clusters were located in North Central and Southeast Florida. These clusters had 15,912 and 19,992 COVID-19 tests per 100,000 persons, and relative risks (RR) of 1.3 and 1.6, respectively (Fig. 3a, Table 2). Low testing rate clusters were located in counties in Northwest, West Central, and East Central Florida. These clusters had 9,072 to 10,237 COVID-19 tests per 100,000 persons and RR of 0.7 to 0.8 (Fig. 3a, Table 2).

**Table 2:** Summary statistics for circular high- or low-risk clusters of number of COVID-19 tests and percent of persons with COVID-19 positive tests

**Outcome variable**	**Cluster type**	**Cluster**	**Cases/100,000**	**Population**	**Observed cases**	**Expected cases**	**No. of counties**	***p*-value**
**Number of COVID-19 tests per 100,000 persons**	High risk	Cluster 1	15,912	2,804,160	447,143	363,599	1	< 0.001
		Cluster 2	19,992	263,753	52,841	34,199	1	< 0.001
	Low risk	Cluster 1	9,137	1,371,878	125,619	177,883	6	< 0.001
		Cluster 2	9,072	669,752	60,889	86,842	6	< 0.001
		Cluster 3	10,237	1,105,034	113,363	143,283	3	< 0.001
**Percent of persons with COVID-19 positive tests**	High risk	Cluster 1	14.2^a^	132,987	18,927	14,573	4	< 0.001
	Low risk	Cluster 1	6.4^a^	331,017	21,276	36,275	32	< 0.001
		Cluster 2	6.5^a^	136,951	8,854	15,008	4	< 0.001
		Cluster 3	6.1^a^	58,436	3,573	6,403	2	< 0.001
		Cluster 4	8.4^a^	62,919	5,316	6,895	2	< 0.001
		Cluster 5	5.4^a^	11,459	624	1,255	1	< 0.001
		Cluster 6	7.9^a^	8,222	651	901	1	< 0.001

^a^Expressed as a percentage

### Positivity rates

The percent of persons with positive tests also varied widely across the state, ranging from 2.7% to 26.6% (Fig. 2b, Table 1). A total of 61 of the 67 (91%) counties in Florida had more than 5% positive tests. Fifty percent of the counties had at least 9% of total persons tested being positive. These counties were concentrated in North Central, Central and Southern Florida regions (Figs. 1 and 2b).

Only one cluster with a high positivity rate (% of positive tests = 14.2%; RR = 1.3) was identified, and it was located in Miami-Dade County in Southern Florida (Fig. 3b). However, FSSS identified 2 additional high positivity rate clusters in Hamilton and Suwanee counties in North Central Florida, and in Martin, Palm Beach, Broward, Miami-Dade, and DeSoto counties in Southern Florida (results not presented). A total of 6 low positivity rate clusters with rates ranging from 6.1—8.4% were identified. The largest low positivity rate cluster was identified in predominantly rural counties in the Panhandle and the Big Bend region. However, an irregularly shaped high positivity rate cluster comprising Hamilton and Suwanee counties was included in that cluster (results not shown). A few other low positivity rate clusters were located in Central East and Southeast Florida (Table 2; Fig. 3b).

### Incidence risks

The median COVID-19 incidence risk was 878 cases per 100,000 persons with wide variations across the state (372 to 3,246 cases per 100,000 persons) (Table 1). The highest incidence risks were concentrated in Southern, Central and a few counties in North Central and Northwest Florida, while the lowest risks were concentrated in Central Florida and the Panhandle region (Fig. 2c).

The geographic distribution of COVID-19 incidence risks overlaps in space with the distribution of testing rates, with areas with the highest testing rates also having the highest incidence risks and vice versa (Figs. 2a, c). However, a number of counties in North Central Florida had the lowest incidence risks (< 705 cases/100,000 persons) but their testing rates were equal to or greater than the median (i.e. 11,697 tests/100,000 persons) testing rate for Florida. On the other hand, the incidence risks for a number of Northwest Florida counties was equal to or above the median incidence risk (878 cases/100,000 persons) for the state (Figs. 2a, b and c) but the testing rates were well below 11,697 tests/100,000 persons.

A total of 4 clusters with high COVID-19 incidence risks were identified (Fig. 3c). Almost all Southern Florida counties, with the exception of Monroe County, were within the high-risk clusters (Fig. 3c). Three small high-risk clusters, each comprising 1—2 counties, were identified in the North Central Florida.

### Percent of hospitalized cases

The percent of hospitalized cases varied from 1.78% to 14.9% (Mean = 7.4%) (Fig. 2, Table 1). The percent of hospitalized cases in both Central and South Florida were above the state average (Fig. 2d).

A total of 4 high-risk clusters of percentage of hospitalized cases were identified. The largest high-risk cluster was located in South Florida and smaller clusters were identified in North Florida (Fig. 3d and Table 3). Additionally, a high-risk cluster of percent of hospitalized cases was identified in Central Florida.

**Table 3:** Summary statistics for circular and non-circular high-risk clusters of number of COVID-19 cases, percent of hospitalized cases, and number of hospitalized cases

**Outcome variable**	**Cluster**	**Cases/100,000**	**Population**	**Observed cases**	**Expected cases**	**No. of counties**	***p*-value**
**Number of COVID-19 cases per 100,000 persons**	Cluster 1	2,211	5,346,797	118,211	75,977	8	0.001
	Cluster 2	2,457	34,126	837	484	2	0.001
	Cluster 3	3,220	8,781	281	124	1	0.001
	Cluster 4	1,709	45,123	771	641	1	0.001
**Percent of hospitalized cases**	Cluster 1	8.4^a^	76,218	6,453	4,962	12	0.001
	Cluster 2	8.8^a^	16,455	1,325	973	10	0.001
	Cluster 3	8.3^a^	11,992	999	778	1	0.001
	Cluster 4	11.0^a^	1,738	190	112	4	0.001
**Number of hospitalized cases per 100,000 persons**	Cluster 1	146	7,727,036	11,249	7,128	12	0.001
	Cluster 2	146	114,177	166	105	3	0.001
	Cluster 3	160	721,053	71	41	1	0.025

^a^Expressed as a percentage

### Hospitalization risks

Hospitalization risks varied from 17 to 282 cases/100,000 (Median = 66) (Fig. 2, Table 1). South Florida had the highest hospitalization risks (> 162 cases/100,000 persons) (Fig. 2e). However, hospitalization risks for the majority of Central Florida and Panhandle counties were below the state median, with Gilchrist County recording the lowest risk (17 cases/100,000) (Fig. 1 and 2e).

A total of 3 clusters of high hospitalization risks were identified. Similar to the clustering patterns observed for percent of hospitalized cases, the largest high-risk clusters were identified in South Florida (Figs. 3d and e) and smaller clusters were identified in North Florida (Fig. 3e and Table 3). Unlike percent of hospitalized cases, no clusters of high hospitalization risks were identified in Central Florida.

### Case fatality rates and mortality risks

There was a clear north–south gradient, with case fatality rates increasing from 0% in the Panhandle counties to 6.3% (median = 1%) in Southern Florida counties (Fig. 2f and Table 1).

Four clusters of high case fatality rates were identified in Florida (Fig. 3f and Table 4). Three of those clusters were located in Southern Florida (Fig. 3f). The relative risks for clusters of high case fatality rates ranged from 1.65 to 4.10 (Fig. 3f).

**Table 4:** Summary statistics for circular and non-circular high-risk clusters of COVID-19 case fatality rates and mortality risks

**Outcome variable**	**Cluster**	**Cases/100,000**	**Population**	**Observed cases**	**Expected cases**	**No. of counties**	***p*-value**
**COVID-19 case fatality rate**	Cluster 1	2.5^a^	50,088	1,257	760	7	0.001
	Cluster 2	2.3^a^	11,992	278	182	1	0.001
	Cluster 3	3.0^a^	3,193	100	51	1	0.001
	Cluster 4	6.6^a^	209	13	3	1	0.016
**Number of COVID-19 deaths per 100,000 persons**	Cluster 1	36	8,060,811	2,870	1,738	10	0.001
	Cluster 2	37	381,071	139	82	1	0.001
	Cluster 3	29	971,022	278	209	1	0.002

^a^Expressed as a percentage

### Mortality risks

The mortality risk varied from 0 to 76 deaths per 100,000 persons (Median = 11.7) across the state (Fig. 2g and Table 1). As with case fatality rates, the mortality risks for Southern Florida counties were above the state median, while those for counties in Central and North Florida were below the state median (Fig. 2g).

The locations of high mortality risk clusters generally mirrored those of high case fatality rates, with all high mortality risks clusters being located in Southern Florida (Figs. 3f, 3g, and Table 4). The relative risks for clusters of high mortality risks ranged from 1.33 to 1.65 (Fig. 3g).

In summary, estimates of all COVID-19 metrics for Southern Florida counties were generally above the state average or median. However, while testing rates, positivity rates and percent of hospitalized cases for a number of rural counties in Northern Florida were above the state average, the incidence risks, hospitalization risks, case fatality rates, and mortality risks for most counties in that region were below the state average. Both circular and irregularly-shaped high-risk clusters for all COVID-19 metrics were concentrated in Southern Florida, with a few clusters of high incidence risks, percent of hospitalized cases, hospitalization risks and case fatality rates in the Northwest and Central Florida. A single high-risk cluster of percent of hospitalized cases was identified in Central Florida. Low testing and positivity rate clusters were concentrated in predominantly rural counties in Northern Florida.

## Discussion

This study retrospectively investigated geographic disparities in COVID-19 testing and positivity rates; incidence, hospitalization and mortality risks; and percent of hospitalized and fatal cases in Florida during the first five months of the pandemic. Study findings will provide useful baseline data to advance our understanding of the spatial dynamics of the pandemic because these findings could be compared to the findings of similar studies using the recent data in Florida. Study findings will also help public health officials gauge the impact of mitigation efforts on the disease burden. Furthermore, Florida’s current age structure and ethnic/racial composition [8] foreshadow the demographic changes projected for the rest of the U.S. by 2050 [18]. Therefore, study findings may provide useful information that may inform response efforts to reduce health disparities across the country.

### Testing rates

Identification of low testing rate clusters in rural counties of Northwest Florida and high testing rate clusters in the predominantly urban Miami-Dade county is consistent with findings from other studies that reported lowest COVID-19 testing rates in rural areas, areas with high poverty levels, and/or large proportions of Black and Hispanic populations, particularly during the early stages of the pandemic [30–34].

The location of COVID-19 testing sites in the U.S. was initially based on the location of existing healthcare infrastructure, which resulted in communities with already low access to healthcare having fewer COVID-19 testing sites per capita [35, 36]. For instance, Tao and co-workers reported that the spatial accessibility to testing sites varied substantially across Florida, with cities having better accessibility and many rural areas being testing deserts [37]. Moreover, the closing of several community health centers and federally qualified health centers during the pandemic left many vulnerable communities without access to timely testing [38, 39]. Thus, the clustering of low testing rates observed in rural counties in Northwest Florida may be attributable to limited access to COVID-19 testing sites in that region. Limited testing capacity, lack of private vehicles, and old age constrained accessibility in a number of densely-populated large cities, resulting in low accessibility in some urban areas as well [37, 40]. Furthermore, minority communities in the more densely-populated U.S. counties/cities had longer travel times for a COVID-19 test than majority-white communities, even after adjusting for median income [35]. Low income black residents were more likely to live in dense urban areas with many testing sites but their accessibility to testing sites was limited by lack of private vehicles [37]. These may account for the low testing rate cluster identified in urban counties in parts of Central Florida.

### Positivity rates

The results of this study showing geographic disparities in positivity rates across Florida are in agreement with those of other ecologic studies [33, 41]. The disparities may be due to differences in social mobility and in the geographic distribution of sociodemographic factors [33, 42–44]. The clustering of high positivity rates in Southern Florida counties suggests widespread infections due to high transmission rates in that community [45]. Due to scarcity of testing resources at existing sites earlier in the pandemic, testing for the SARS-CoV-2 virus was largely limited to diagnostic testing of symptomatic persons [34, 37, 46]. Thus, clustering of high positivity rates may also be attributable to exclusive testing of more advanced cases, thereby leaving out mild and asymptomatic cases, particularly in younger patients [47, 48]. The clustering of low positivity rates in Northern Florida counties, on the other hand, suggests low rates of coronavirus transmission relative to the amount of testing in those counties.

### Incidence risks

High-density living settings and high population density have been associated with high SARS-CoV-2 infection rates [49–52]. Consequently, low population densities in predominantly rural Northern Florida counties may account for the clustering of low COVID-19 incidence risks in that region. Additionally, lack of transportation and low availability of testing sites might have negatively affected the number of rural residents tested for SARS-CoV-2 [37, 53], leading to underreporting of cases, which in turn contributed to the observed clustering of low incidence risks in rural counties of the north.

Southern Florida, on the other hand, has large proportions of Hispanic and non-White immigrant populations, a large proportion of whom live in multigenerational overcrowded households in densely populated areas [52, 54–60]. These groups are also more likely to rely on public or shared modes of transportation, and to work in low-income, essential jobs in public-facing sectors that make it difficult or impossible to adopt COVID-19 preventive measures such as telecommuting and social distancing [61–63]. These conditions favor connectivity and higher contact rates, potentially increasing exposure level of the population to the virus [57, 64, 65], thereby resulting in higher COVID-19 incidence risks in counties with large proportions of these communities [66, 67]. Thus, the clustering of high COVID-19 risks in Southern Florida likely reflects higher infection rates among Hispanic/Latino and foreign-born residents due to higher risks of exposure to the SARS-COV-2 virus at work as well as crowded living and transportation settings. Similar to the findings of this study, hotspots of high COVID-19 risks were reported for areas with large household sizes and/or high concentrations of service workers and socioeconomically disadvantaged minority groups in Chicago and New York [58, 68]. Low socioeconomic status [67, 69, 70] may also have contributed to higher SARS-CoV-2 virus transmission rates and hence clustering of high COVID-19 risks in that region.

It has been postulated that a high incidence of COVID-19 in certain areas could be due to high testing rates in those areas [71]. However, this is not consistent with the findings of the current study that revealed: (i) clustering of high COVID-19 incidence risks but not of high testing rates in a number of Northern and Southern Florida counties and (ii) clustering of high testing rates but not of high incidence risks in Alachua County.

### Hospitalization and mortality risks

The identified high-risk hospitalization and mortality clusters in this study parallel reports from other studies examining placed-based inequities in these COVID-19 outcomes both in the US [50, 67, 68, 72] and other countries [73, 74].

The clustering of high COVID-19 hospitalization and mortality risks in Southern Florida may be a reflection of the disproportionate burden of COVID-19 borne by Black and non-white Hispanic/Latino communities [75, 76]. These communities are particularly vulnerable to severe illness and death due to higher rates of medical risk factors for COVID-19 morbidity and mortality, such as diabetes mellitus, obesity, hypertension, cardiovascular disease and lung disease [77–81]; high rates of tobacco smoking [82]; limited access to high quality healthcare [34, 83, 84]; and higher levels of ambient pollution, such as NO_2_ and PM 2.5, due to residence in close proximity to sources of air pollution and longer distance from regulatory air quality monitoring compared to white communities [85–87]. This is in spite of substantial reductions (~ 50%) in passenger vehicle traffic in the least white urban communities during the lockdown period [87]. These factors are positively associated with increased severity and risk of adverse COVID-19 outcomes [88–93]. Socioeconomic factors such as high poverty and limited access to healthy foods, household- and county-level overcrowding [74, 94–96] may also have contributed to clustering of adverse COVID-19 outcomes in Southern Florida. In addition, counties in Southern Florida have higher burden of older population (≥ 65 years old) compared to counties in Northern Florida [97]. Since older individuals are more likely to have two or more comorbidities, they have high risk of adverse COVID-19 outcomes. It has been reported that persons age 65 years or older had strikingly higher COVID-19 mortality rates compared to younger individuals [98]. Therefore, high proportion of older population contribute to high-risk clusters of hospitalization and mortality in Southern Florida.

### Percent of hospitalized cases and case fatality rate

The fact that Miami-Dade, Collier, and Lee Counties were not included in the high-risk cluster in Southern Florida when hospitalized cases were divided by total cases, but the converse was true when incident and hospitalized cases were divided by county population suggests that a large proportion of the incident cases were not severe enough to warrant hospitalization. These urban counties have a relatively younger population, implying that a large proportion of the cases were less susceptible to severe disease compared to the rest of Florida. This explanation is bolstered by the absence of clusters of high case fatality rates in those counties. Surprisingly, the opposite trend was observed for St. Lucie County, which is also an urban county in Southeast Florida. Potential reasons for high susceptibility to more adverse COVID-19 outcomes in St. Lucie County include high percentage of individuals living below the federal poverty limit, low healthcare accessibility, and high burden of comorbidities. Not surprisingly, the average life expectancy in St. Lucie is consistently lower than that for Miami-Dade, Collier, and Lee counties [99]. The clustering of high case fatality rates in several Southern Florida countries may be due to limited access to healthcare facilities with intensive care unit (ICU) beds in those counties [84]. Many areas in Southern Florida, particularly those with large Latino or Hispanic populations, were reported to be more likely to have lower access to resources for critical healthcare compared to other regions during the COVID-19 pandemic [83, 84].

The identification of high-risk clusters in Calhoun, Gadsden, and Jackson counties in Northwest Florida both when hospitalized cases were -divided by the total number of cases and by the county population suggests that despite the lower COVID-19 incidence risks in Northwest Florida compared to Southern Florida, a large proportion of the cases in Northwest Florida were severe enough to warrant hospitalization. Northwest Florida is a predominantly rural region with large proportions of black and older residents, and high burdens of comorbidities such as diabetes, hypertension and cardiovascular disease [81, 100–102]. Moreover, similar to what was reported for black patients in California [103], limited access to outpatient testing sites in Northwest Florida [37] may result in a large proportion of black segments of the population accessing testing in hospitals when symptoms are already severe. These, coupled with low access to healthcare and low socioeconomic status or high social vulnerability [83, 104] are associated with higher risks of more severe COVID-19 outcomes [68, 88, 105], and they may account for clustering of high hospitalization risks and percent of hospitalized cases in those counties.

The identification of a high-risk cluster of percent of hospitalized COVID-19 cases in parts of Central Florida around Gainesville may be due to higher level of accessibility of healthcare facilities equipped with ICU beds in that region [83, 84]. Additionally, this being a predominantly urban area, the median household incomes, health insurance rates, proportion of younger (< 65 years) residents, education attainment levels, and socioeconomic status are generally higher than those for rural areas [104]. These factors, coupled with higher quality of healthcare and a higher tendency for urban residents to seek healthcare may increase healthcare utilization rates [106, 107], and contribute to clustering of high percent of COVID-19 hospitalization risks in North Central Florida. Furthermore, the lack of clustering of high case fatality rates in parts of North Central Florida provides further evidence that access to healthcare is the most likely explanation for clustering of high percent of hospitalized cases in that region.

### Incidence, hospitalization, and mortality risks in rural Florida

The identification of low-risk clusters of percent of positive tests and only a few high-risk clusters of incidence, hospitalization, and mortality risks in rural counties in Northern Florida suggests that the impact of COVID-19 was lower in rural areas than in urban areas during the study period. These results are consistent with those of other area-level studies reporting lower COVID-19 incidence and deaths in the most socioeconomically vulnerable counties during the early stage of the pandemic [108–113]. The lower COVID-19 incidence and hospitalization and risks in rural Florida counties may be attributed to low population density in those counties [114] which resulted in lower contact rates thereby decreasing opportunities for transmission of the SARS-CoV-2 virus [56, 57, 115]. Additionally, preventive measures that limited movement and social interactions may also have contributed to low COVID-19 infection risks in the already sparsely populated rural counties [116]. However, lower access to testing in under resourced rural counties with a high prevalence of risk factors may lead to fewer deaths attributable to COVID-19 and bias results towards the null.

It is worth noting that despite reduced opportunities for the SARS-COV-2 virus to spread in the sparsely populated rural Florida counties, these locales have, on average, larger proportions of older (> 65 years) residents and higher burdens of underlying chronic health conditions such as obesity [117] diabetes [81, 101], hypertension [100], and heart disease [102, 118, 119] compared to urban counties. Rural areas also tend to have low health insurance rates and a less robust healthcare infrastructure [120–123]. All of these factors make rural communities uniquely vulnerable to severe disease and death from COVID-19 [124]. Thus, similar to other studies showing substantially higher COVID-19 incidence and death risks in U.S. metro/urban areas compared to nonmetro/rural areas earlier in the pandemic but higher risks of these in the latter stages of the pandemic [108–112], rural Florida counties may have disproportionately higher COVID 19 incidence and death risks compared to their urban counterparts in the later stages of the pandemic. Northwest Florida is particularly vulnerable to poorer outcomes due to a shortage of healthcare resources with capacity to handle a large number of cases [83, 125].

### Study strengths and limitations

The use of rigorous spatial epidemiological tools (FSSS and CSSS) coupled with the investigation of disparities in COVID-19 testing and positivity rates and several of its outcomes enabled this study to obtain a more comprehensive picture of the burden of COVID-19 in Florida during the early stage of the pandemic. The FSSS method identified irregularly shaped local clusters that would otherwise be missed by the CSSS method, which is used by most studies for identifying local spatial clusters. A limitation of the study is the potential for an underreporting of COVID-19 incidence, hospitalization, and mortality risks and an overestimation of case fatality rates. This is because the number of cases diagnosed was contingent on local SARS-CoV-2 testing availability and guidelines, which varied across the state and evolved during the study period as the understanding of COVID-19 clinical presentations and its risk factors improved. Additionally, we did not identify the contextual factors that may be associated with the identified disparities. Therefore, our discussion is based on our knowledge of the underlying population groups in areas with high COVID-19 incidence and death risks and low testing. Identifying determinants may aid in the development of novel approaches to reduce the disproportionate burden of COVID-19 in some populations to achieve greater health equity. Additionally, the FSSS analysis failed to run when we attempted to detect high testing rate clusters, hence it is likely that irregularly shaped clusters were missed.

Finally, aggregating data by county may obscure substantial local heterogeneities that may be present at a lower spatial scale [126, 127]. Thus, an analysis at a lower geographic level, such as the Zip Code or Census Tract, may provide a more comprehensive picture of the COVID-19 burden and enable more efficient targeting of place-based initiatives.

## Conclusion

Substantial geographic disparities in COVID-19 testing and positivity rates; incidence, hospitalization and mortality risks; and percent of hospitalized and fatal cases exist across Florida early in the pandemic, with counties in Southern Florida generally having higher testing rates and poorer outcomes compared to those in the Northern Florida. These findings may provide baseline data to evaluate the effectiveness of interventions aimed at controlling the pandemic and reducing health disparities in Florida. Future studies will need to assess changes in spatial patterns over time at lower geographical scales and determinants of any identified patterns.

## Supplementary Information

**Additional file 1:** **Supplementary File 1.** Raw COVID-19 data for Florida counties, March 1 to July 15, 2020.

## Data Availability

All study data have been included in the submission.
